# Repeat Meningitis/Encephalitis Syndromic Panel Testing After an Index-Negative Result: A Retrospective Laboratory-Record Study

**DOI:** 10.3390/microorganisms14102310

**Published:** 2026-10-10

**Authors:** Jeong Su Han, Pil Seung Kwon, Jae-Sik Jeon, Jae Kyung Kim

**Affiliations:** 1 Department of Biomedical Laboratory Science, College of Health Sciences, Dankook University, Cheonan-si 31116, Republic of Korea; jshan1162@naver.com (J.S.H.); zenty87@naver.com (J.-S.J.); 2 Department of Biomedical Laboratory Science, College of Healthcare and Medical Sciences, Wonkwang University, 514 Iksan-daero, Iksan-si 54538, Republic of Korea; pskwon@wu.ac.kr

**Keywords:** meningitis/encephalitis panel, multiplex PCR, cerebrospinal fluid, repeat testing, laboratory utilization, molecular diagnostics

## Abstract

Syndromic molecular panels rapidly detect predefined central nervous system pathogens, but the incremental microbiological yield of repeating the same panel after an index-negative result is uncertain. We retrospectively analyzed 313 meningitis/encephalitis (ME)-panel episodes from 265 patients from September 2023 to March 2026. Among 228 patients with a first-observed index-negative panel, 30 underwent subsequent repeat testing and 198 had no subsequent repeat ME-panel test recorded at this institution during the study period. Repeat-tested patients had higher index-panel-linked cerebrospinal fluid (CSF) white blood cell (WBC) counts (median 28.0 vs. 4.0 cells/µL; *p* < 0.001) and protein concentrations (73.4 vs. 52.4 mg/dL; *p* = 0.004), whereas glucose did not differ significantly. In the 30 repeat-tested index-negative patients, no negative-to-positive conversion was observed (0/30; exact 95% confidence interval, 0.0–11.6%), and none of the 44 repeat tests detected a new on-panel target. Among 28 persistently negative patients with paired CSF WBC results, the median count decreased from 30.5 to 3.5 cells/µL. Clinical indications, treatment exposure, immune status, and final diagnoses were unavailable. These findings provide laboratory context for repeat testing but do not determine patient-level clinical appropriateness or exclude central nervous system infection.

## 1. Introduction

Acute meningitis and encephalitis require timely etiologic assessment because infectious causes span viral, bacterial, and fungal pathogens with divergent treatment implications. Rapid syndromic molecular panels can identify a predefined set of central nervous system (CNS) pathogens within approximately one hour and may support earlier targeted management [1,2,3]. Independent evaluations using clinical specimens have confirmed high agreement for most panel targets while documenting pathogen-specific limitations [4]. Their broad availability, however, also raises questions about real-world test utilization, interpretation, and repeat ordering [5,6,7]. Once such assays are embedded in routine care, the frequency, timing, and outcomes of repeat ordering become epidemiological features of test use and diagnostic stewardship, distinct from the diagnostic accuracy of a single run.

A negative meningitis/encephalitis (ME)-panel result narrows but does not eliminate the differential diagnosis. The assay is restricted to predefined targets, and a negative result may coexist with off-panel infection [8], low organism burden, sampling-timing effects, prior treatment, or noninfectious inflammation such as autoimmune encephalitis [9]. Herpes simplex virus DNA, for example, may be undetectable in cerebrospinal fluid (CSF) obtained very early in the clinical course [10], which is one of the settings in which repeat molecular testing has an established rationale [11]. In a consecutive series of 1000 patients, 84 patients received a diagnosis of CNS infection despite a negative ME-panel result; among panel-negative patients without a CNS infection, common alternative diagnoses included primary headache disorders, cranial nerve palsies, neuroinflammatory disorders, and seizures [12]. Repeat lumbar puncture and repeat molecular testing may therefore occur when neurologic symptoms persist, when the initial specimen is considered suboptimal, or when the diagnostic hypothesis changes [13,14,15]. Accordingly, a persistently negative syndromic-panel result should not be equated with the absence of CNS infection.

Prior studies have mainly evaluated diagnostic accuracy, overall positivity, clinical impact, and testing algorithms [1,2,3,4,5,6,16,17]. A recent real-world syndromic multiplex-PCR utilization study identified and removed repeat determinations from the same patient using the same panel before analysis, illustrating that longitudinal repeat sequences are often excluded from routine utilization cohorts [18]. Published repeat-testing evaluations of the ME panel have addressed technical repetition of the same specimen after invalid, discordant, or multiple-target results, in which most repeated results were not reproducible [19]. Reordering the panel on a newly collected specimen after an index-negative result is a different laboratory action, and its frequency, timing, and additional on-panel detection yield have received little patient-level longitudinal evaluation. Reordering the same qualitative molecular panel and reassessing the evolving cellular and biochemical CSF profile are related but noninterchangeable laboratory actions.

We conducted a retrospective longitudinal laboratory cohort study of real-world ME-panel use in a tertiary hospital. The aims were to characterize the frequency and timing of repeat testing; describe available laboratory characteristics according to subsequent repeat-testing status among patients with a first-observed index-negative result; estimate patient-level negative-to-positive conversion after an index-negative result; describe chronologically ordered within-institution panel-result sequences; and evaluate within-patient changes in CSF WBC count, protein, and glucose among persistently negative sequences. The central microbiological question was deliberately narrow: whether repeating the same predefined syndromic target set produced additional on-panel detection. Repeat panel ordering, serial CSF reassessment, and targeted or off-panel microbiological investigation were therefore treated as conceptually distinct diagnostic actions.

## 2. Materials and Methods

### 2.1. Study Design and Setting

This retrospective, single-center observational study used de-identified laboratory information system data from a tertiary-care university hospital in the Republic of Korea. The study period extended from 3 September 2023, when data required for the present analysis became available, to 30 March 2026. All age groups were eligible; age was recorded in completed years, and an age of 0 denoted an infant younger than 1 year.

The analytic dataset comprised all 313 eligible ME-panel episodes from 265 patients available during the study period. Because this was a retrospective census of all eligible records within the defined study period, the sample size was determined by data availability rather than a target effect size; no a priori sample-size or power calculation was performed. One adjudicated same-day duplicate negative polymerase chain reaction (PCR) episode, linked to the same CSF specimen as an earlier PCR episode, was excluded. Patients were eligible for the repeat-testing analysis when at least two distinct panel episodes were recorded under the same patient identifier during the study period. CSF specimens were processed and tested according to the manufacturer’s instructions and the laboratory’s validated routine standard operating procedures.

All ME-panel and CSF tests were performed as part of routine clinical care, and research-specific data extraction and statistical analysis were initiated only after institutional review board approval. The study was reported in accordance with the STROBE and RECORD statements for observational and routinely collected health-data studies [20,21]. The analytical source contained de-identified laboratory records only, and no additional chart review was performed for the present study. Accordingly, clinical indications for repeat testing, symptom onset or trajectories, antimicrobial or antiviral exposure, immune status, imaging, culture, targeted or off-panel microbiological results, final diagnoses, and outcomes were not available in the analytical extract.

The institutional archive partially overlaps with datasets used in separate studies addressing nonoverlapping hypotheses and outcomes; no repeat-testing sequence or serial first-to-last CSF analysis reported here was published or submitted elsewhere.

### 2.2. ME-Panel Episodes and Repeat-Testing Sequences

A panel episode was defined as one reportable BioFire FilmArray^®^ Meningitis/Encephalitis (ME) panel (FA/ME; BioFire Diagnostics, bioMérieux LLC, Salt Lake City, UT, USA) run represented by a unique episode record. The assay was performed using the BioFire FilmArray^®^ platform (BioFire Diagnostics, LLC, Salt Lake City, UT, USA). The ME-panel assay was used within the laboratory’s validated performance specifications and was subject to routine internal quality-control procedures. A repeat test was defined as an ME panel performed on a newly collected CSF specimen with a distinct specimen-collection timestamp and specimen or accession identity. Technical reruns, reruns following an invalid or error result, and duplicate records linked to the same CSF specimen were not classified as independent repeat tests. Only valid final results released to the laboratory information system were included.

As a qualitative assay, results were classified as positive when at least one of the 14 panel targets was detected and negative when no target was detected. The 14 predefined bacterial, viral, and fungal targets are listed in Supplementary Table S2. Episodes were ordered chronologically within each patient using the PCR specimen-collection timestamp. The earliest panel recorded during the study period was designated the index observed panel, hereafter the index panel; all subsequent panels were classified as repeat tests. This definition denotes the first panel observed within the study window and does not assume that it was the patient’s first lifetime ME panel or the first panel for the presenting illness. Detected targets among all positive episodes were summarized by pathogen class and according to whether positivity occurred on the index panel or a repeat panel. Single- and multiple-target detections were also assessed.

Repeat testing was not governed by a study protocol; all clinically ordered repeat panels meeting the above laboratory definition were included. The clinical criteria used by individual physicians to select patients for repeat testing were not available in the laboratory dataset. Repeat-testing status reflected only ME-panel tests recorded at this institution during the study window. Repeat-testing frequency was calculated as the proportion of unique patients who underwent at least one repeat panel during the study period. Negative-to-positive conversion was evaluated primarily at the patient level among patients whose index panel was negative; the number of repeat tests yielding a new positive result was reported descriptively because multiple repeat tests within one patient are not independent observations. The persistently negative longitudinal cohort comprised patients whose index panel was negative and who had no positive result anywhere in their available within-institution sequence. Intervals from the index panel to the first repeat panel, between consecutive panels, and from the index to the final panel were calculated from specimen-collection timestamps. Because widely separated tests could represent distinct clinical events rather than repeat testing within the same illness episode, negative-to-positive conversion was re-evaluated in sensitivity analyses restricted to repeat panels performed within 14 and 30 days of the index panel.

### 2.3. CSF Measurements and Linkage of Cellular and Biochemical Data

CSF specimens were analyzed promptly after receipt; specimens requiring delayed testing were stored at 2 °C to 8 °C in accordance with the laboratory’s validated SOP. CSF WBC, protein, and glucose measurements were performed as part of routine clinical care using laboratory-validated procedures and were subject to routine internal quality-control processes. Values were extracted from final validated laboratory reports; no research-specific remeasurement was performed. CSF WBC counts were determined by manual microscopy using a Neubauer counting chamber (BRAND GmbH + Co KG, Wertheim, Germany) without staining and were reported as cells/µL. Because cells/µL is numerically equivalent to ×10^6^/L, WBC is presented in cells/µL throughout the text; SI values are given in parentheses for protein (g/L) and glucose (mmol/L).

Reported, uncorrected CSF WBC values were used in the primary longitudinal analysis. CSF total protein was measured using the Total Protein Urine/CSF Gen.3 assay (TPUC3), a benzethonium chloride turbidimetric method, and CSF glucose using the hexokinase/glucose-6-phosphate dehydrogenase method, both on the c 702 module of a cobas^®^ 8000 modular analyzer series (Roche Diagnostics GmbH, Mannheim, Germany), with manufacturer-supplied calibrators and control materials. Reagent lot- and calibration-level records were not retained in the de-identified analytical extract.

Laboratory records were reconciled at the specimen level using patient identifiers, specimen or accession identifiers, and specimen collection and receipt timestamps. When a shared specimen or accession identity was available, it was prioritized. If a direct cross-file identifier was unavailable, candidate links were generated using the same patient identifier and temporal proximity and then manually reviewed against collection and receipt times, analyte availability, and within-patient test order. The nearest eligible CSF cellular specimen within 48 h was retained; a link was not accepted on temporal proximity alone when the available identifiers or test sequence were discordant. The 48-h window accommodated asynchronous laboratory timestamps, and robustness was assessed using a 24-h linkage restriction. Linkage quality was summarized using the absolute PCR–CSF interval, the proportions linked within 12 and 24 h and on the same calendar day, and the direction of the signed interval. CSF chemistry was linked to the selected cellular specimen when available within 12 h of the cellular-specimen receipt time. During data validation, CSF protein and glucose variable labels were cross-checked against the validated laboratory variable mapping and corrected before analysis; the underlying measurements were not modified. No missing values were imputed.

### 2.4. Outcomes and Statistical Analysis

The primary outcome was the observed patient-level incremental on-panel target-detection yield, defined as negative-to-positive conversion after an index-negative panel. This outcome was restricted to additional detection among the 14 predefined panel targets and was not used to classify CNS infection as present or absent. Exact Clopper–Pearson 95% confidence intervals (CIs) were calculated for patient-level zero-event proportions, and zero-event results were interpreted in accordance with established guidance on zero numerators [22]. Because the primary analysis was based on a fixed retrospective cohort and yielded zero conversion events, precision was characterized using the exact 95% CI rather than a post hoc power calculation. Repeat-test-level event counts were reported descriptively without binomial CIs because multiple repeat tests within the same patient were not independent. Overall episode positivity, repeat-testing frequency, and patient characteristics according to single versus repeat testing were summarized descriptively. As a post hoc exploratory analysis providing laboratory context for subsequent repeat testing, all patients with a first-observed index-negative ME-panel result were classified according to whether a subsequent repeat ME-panel test was recorded at this institution during the study period. Age, sex, and available CSF WBC, protein, and glucose measurements linked to the first-observed index panel were compared between patients with and without a recorded subsequent repeat test. For this comparison, measurements linked only to subsequent repeat panels were not substituted when an index-panel-linked value was unavailable. The same PCR–CSF and cellular–chemistry linkage definitions used elsewhere in the study were applied. Continuous variables were compared using two-sided Wilcoxon rank-sum tests and sex using Fisher’s exact test. Because this five-variable comparison was post hoc and exploratory, Benjamini–Hochberg false-discovery-rate (FDR) adjustment was additionally applied across the five comparisons as a multiplicity sensitivity analysis. These comparisons were not intended to identify clinical indications for repeat testing.

For each CSF marker, patients with at least two nonmissing linked measurements were included, and the first and last available linked values were paired within patients. Continuous variables were summarized as medians and interquartile ranges (IQRs), with quartiles calculated using the type 7 sample-quantile definition implemented in R software (version 4.3.3; R Foundation for Statistical Computing, Vienna, Austria). First and last linked CSF values were compared using two-sided Wilcoxon signed-rank tests with asymptotic *p* values and no continuity correction. Within the primary analysis and sensitivity analyses evaluating all three CSF markers, *p* values across the three marker comparisons were adjusted using the Benjamini–Hochberg false-discovery-rate (FDR) procedure. Median paired changes were accompanied by percentile bootstrap 95% CIs based on 20,000 patient-level resamples. Bootstrap CIs and Wilcoxon signed-rank tests were interpreted as addressing different statistical quantities. Direction of change was classified as decreased, unchanged, or increased. Post hoc sensitivity analyses included restriction of negative-to-positive conversion to repeat tests occurring within 14 and 30 days of the index panel; repetition of paired CSF-marker analyses using a 24-h PCR–CSF linkage window; comparison of CSF values linked to the index panel and the first repeat panel; and first-to-last comparisons restricted to complete observed panel sequences occurring within 30 and 14 days. Within-patient direction of change and Benjamini–Hochberg-adjusted q values were reported for each marker analysis. The same paired Wilcoxon signed-rank test and 20,000-resample patient-level percentile bootstrap procedure were applied to a sensitivity analysis excluding patients with a cerebrospinal fluid red blood cell (RBC) count >500 cells/µL (>500 × 10^6^/L) in either the first or last paired specimen. This laboratory threshold was used only as a sensitivity-analysis criterion and was not treated as a definitive clinical classification of a traumatic lumbar puncture. Because this analysis comprised a single targeted WBC comparison, FDR adjustment was not applied.

**Figure 3:**
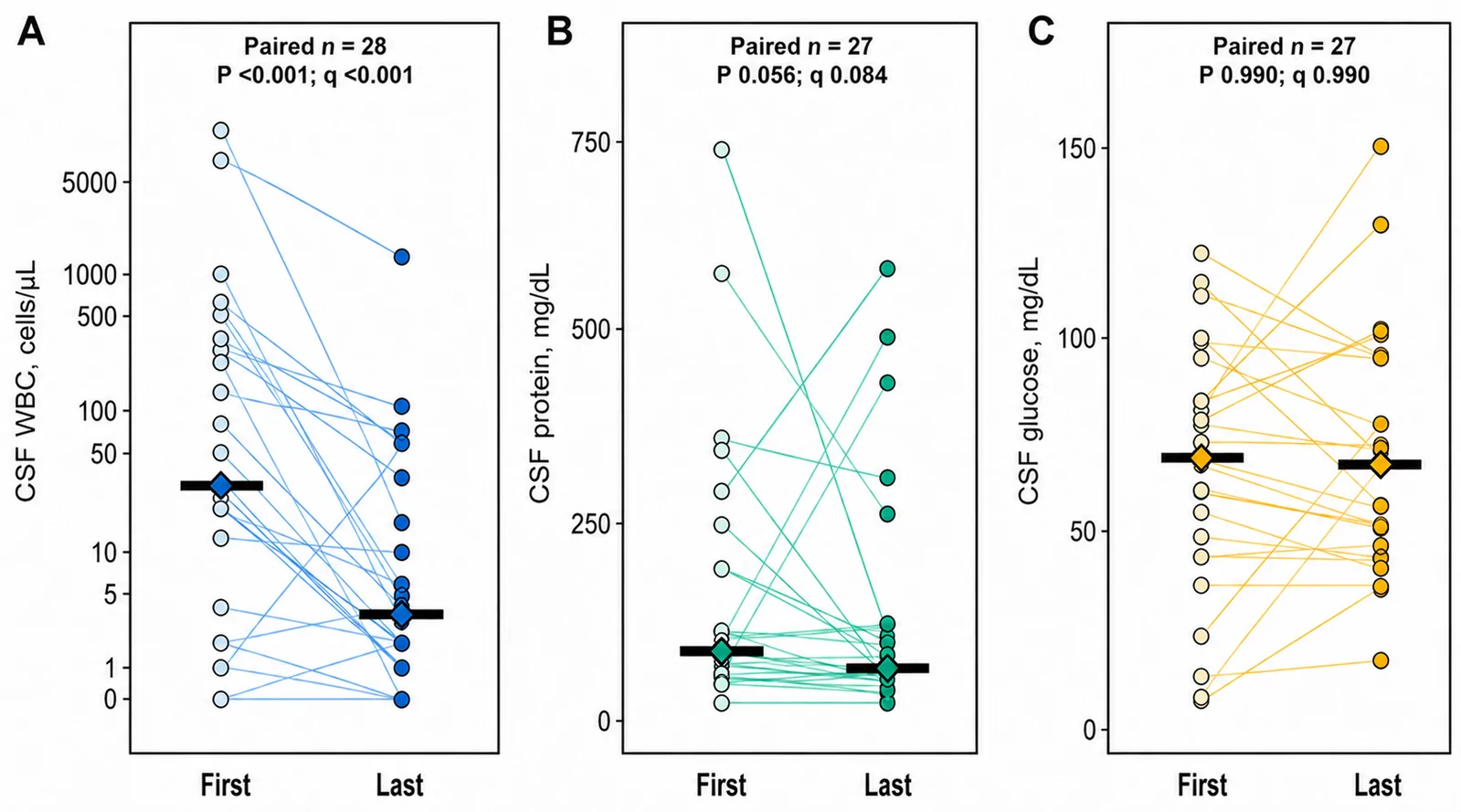
Exploratory within-patient changes in CSF cellular and biochemical markers during persistently negative ME-panel sequences. Paired first and last measurements are shown for (**A**) CSF white blood cell count, (**B**) CSF protein, and (**C**) CSF glucose. Each line connects measurements from the same patient; light circles indicate the first measurement, dark circles the last, and diamonds with horizontal bars the median at each time point. For visualization only, the CSF WBC axis used a pseudo-logarithmic transformation with base 10 and sigma 1 to accommodate zero values and the right-skewed distribution; statistical analyses used untransformed values. *p* values were calculated using two-sided Wilcoxon signed-rank tests and q values were calculated using Benjamini–Hochberg FDR correction. Axes show conventional units. Corresponding SI values are reported in Table 4; for conversion, cells/µL is numerically equivalent to ×10^6^/L, protein mg/dL × 0.01 = g/L, and glucose mg/dL × 0.0555 = mmol/L. Abbreviations: CSF, cerebrospinal fluid; ME, meningitis/encephalitis; WBC, white blood cell count. Alt text: Paired first-to-last CSF measurements among persistently ME-panel-negative patients. White blood cell counts decrease in most paired patients and are statistically significant after false-discovery-rate correction; protein shows a nonsignificant primary decrease and glucose shows no significant change.

The same paired testing and bootstrap procedures were applied separately within each analysis. Because the sensitivity analyses used overlapping subsets of the same patients, they were interpreted as robustness checks rather than independent replications; Benjamini–Hochberg correction was applied separately within, not across, analyses. Figure 3 for the CSF-marker analyses, statistical significance was assessed using an FDR-adjusted q value of <0.05; raw two-sided *p* values are also reported. Analyses were performed using R version 4.3.3 (R Foundation for Statistical Computing, Vienna, Austria). No novel software was developed; the analysis scripts are available from the corresponding author upon reasonable request. During manuscript preparation, the authors used ChatGPT-5.6 (OpenAI, San Francisco, CA, USA) solely for English-language editing to improve grammar, clarity, and readability. The authors reviewed and edited all output and take full responsibility for the final content. The tool was not used for study design, data collection, statistical analysis, or interpretation of the results.

### 2.5. Ethics

The study was conducted in accordance with the Declaration of Helsinki and approved by the Institutional Review Board of Dankook University Hospital (protocol 2026-06-023-003; approved 24 July 2026). The requirement for informed consent was waived because the study used de-identified routinely collected laboratory data.

## 3. Results

### 3.1. Cohort Composition and Repeat-Testing Characteristics

The full analytic cohort comprised 313 ME-panel episodes from 265 patients, of which 38 episodes (12.1%) were positive. Of the 38 positive episodes, 32 (84.2%) contained a viral target and six (15.8%) contained a bacterial target; no fungal or multiple-target detections were identified. The most frequently detected targets were varicella-zoster virus (12/38, 31.6%), enterovirus (8/38, 21.1%), and herpes simplex virus 2 (7/38, 18.4%) (Supplementary Table S2). At each patient’s first observed panel, the median age was 38.0 years (IQR, 1.0–64.0), and 157 patients (59.2%) were male. First-observed panels were negative in 228 patients (86.0%) and positive in 37 (14.0%). Most patients (231/265, 87.2%) were tested once, whereas 34 patients (12.8%) underwent repeat testing, contributing 82 episodes and 48 repeat tests (48/313 episodes, 15.3%).

**Figure 1:**
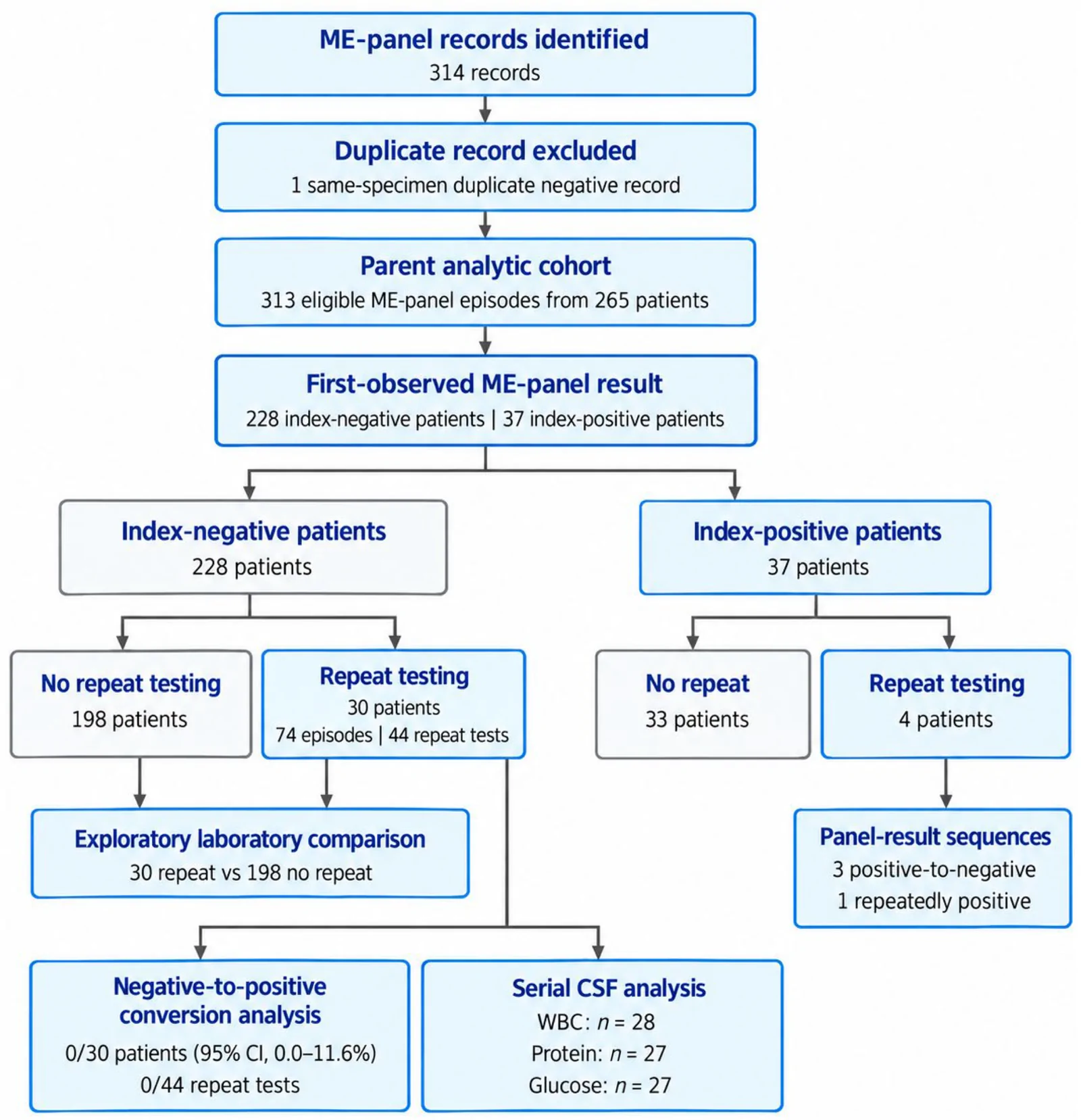
Derivation of the analytic cohort and allocation according to the first-observed ME-panel result. Of 265 patients, 228 had a first-observed index-negative panel and 37 had an index-positive panel. Among index-negative patients, 198 had no subsequent repeat ME-panel test recorded at this institution during the study period and 30 underwent repeat testing, contributing 74 episodes and 44 repeat tests. The exploratory laboratory comparison used the full index-negative cohort (30 repeat vs. 198 with no repeat recorded), whereas the primary negative-to-positive conversion analysis was necessarily restricted to the 30 repeat-tested patients (0/30; exact 95% CI, 0.0–11.6%). Among 37 index-positive patients, 33 had no subsequent repeat test recorded and four underwent repeat testing; three changed from positive to negative and one remained positive for the same target. In the flow diagram, “No repeat testing” denotes no subsequent ME-panel test recorded at this institution during the study period. Abbreviations: CI, confidence interval; CSF, cerebrospinal fluid; ME, meningitis/encephalitis.

Patients undergoing repeat testing had a median age of 55.5 years (IQR, 40.5–58.8), and 26 of 34 (76.5%) were male, compared with 34.0 years (IQR, 0.0–64.0) and 131 of 231 (56.7%), respectively, among patients tested once. Repeat-tested patients were older and more frequently male than patients tested once; these descriptive differences were not interpreted as predictors of repeat ordering. Among the 228 patients with a first-observed index-negative panel, 198 had no subsequent repeat ME-panel test recorded at this institution during the study period and 30 underwent repeat testing; among the 37 patients with a first-observed index-positive panel, 33 had no subsequent repeat test recorded and four underwent repeat testing (Figure 1).

Among all 34 repeat-tested patients, 26 underwent two tests, five underwent three tests, and one patient each underwent four, five, and six tests. The median interval from the index panel to the first repeat panel was 7.65 days (IQR, 5.52–11.48; range, 0.36–81.77), the median interval between consecutive panels was 7.30 days (IQR, 6.03–11.40; range, 0.36–81.77), and the median interval from the index to the final panel was 9.03 days (IQR, 6.62–14.31; range, 1.91–93.32).

Overall, 31 of 48 repeat tests (64.6%) were performed within 14 days and 40 of 48 (83.3%) were performed within 30 days of the index panel. Within the persistently negative cohort (*n* = 30), 22 patients underwent two tests, five underwent three tests, and one patient each underwent four, five, and six tests. The median age at the index panel was 54.5 years (IQR, 40.5–62.0), and 23 patients (76.7%) were male. The median interval from the index panel to the first repeat panel was 7.30 days (IQR, 5.16–12.33), between consecutive panels it was 7.14 days (IQR, 5.87–11.52), and from the index to the final panel it was 8.41 days (IQR, 6.62–14.83) (Table 1).

### 3.2. Laboratory Characteristics According to Subsequent Repeat Testing Among Index-Negative Patients

Among the 228 patients with a first-observed index-negative panel, 30 subsequently underwent repeat testing and 198 had no subsequent repeat ME-panel test recorded at this institution during the study period. Patients who underwent repeat testing were older (median [IQR], 54.5 [40.5–62.0] vs. 32.0 [0.0–64.0] years; *p* = 0.047) and more frequently male (23/30 [76.7%] vs. 110/198 [55.6%]; *p* = 0.030). Among patients with available index-panel-linked CSF results, the repeat-tested group had higher CSF WBC counts (28.0 [8.5–262.0] vs. 4.0 [1.0–15.0] cells/µL; *p* < 0.001) and higher CSF protein concentrations (73.4 [46.7–160.0] vs. 52.4 [29.5–79.1] mg/dL; *p* = 0.004), whereas CSF glucose did not differ significantly (70.0 [49.5–82.0] vs. 60.0 [50.0–73.0] mg/dL; *p* = 0.271). In the Benjamini–Hochberg multiplicity sensitivity analysis across these five post hoc comparisons, CSF WBC (q < 0.005) and protein (q = 0.010) remained below q < 0.05, whereas age (q = 0.059), sex (q = 0.0504), and glucose (q = 0.271) did not. These comparisons describe the observed laboratory context of subsequent repeat testing and were not interpreted as identifying the clinical reason for reordering (Table 2).

### 3.3. Incremental On-Panel Target-Detection Yield and Panel-Result Sequences

The primary patient-level analysis included 30 repeat-tested patients with an index-negative panel. No negative-to-positive conversion was observed (0/30 patients; 0.0%; exact 95% CI, 0.0–11.6%). Across these patients, none of 44 repeat tests yielded a new on-panel target detection. The findings were unchanged when testing was restricted to temporally proximate repeats. No patient-level conversion was observed within 14 days [0/25 patients; exact 95% CI, 0.0–13.7%] or within 30 days [0/28 patients; exact 95% CI, 0.0–12.3%]. At the descriptive repeat-test level, no new target was detected in 27 repeat tests within 14 days or in 36 repeat tests within 30 days. All patients with an index-negative panel therefore remained negative across their observed institutional sequences (Table 3).

**Figure 2:**
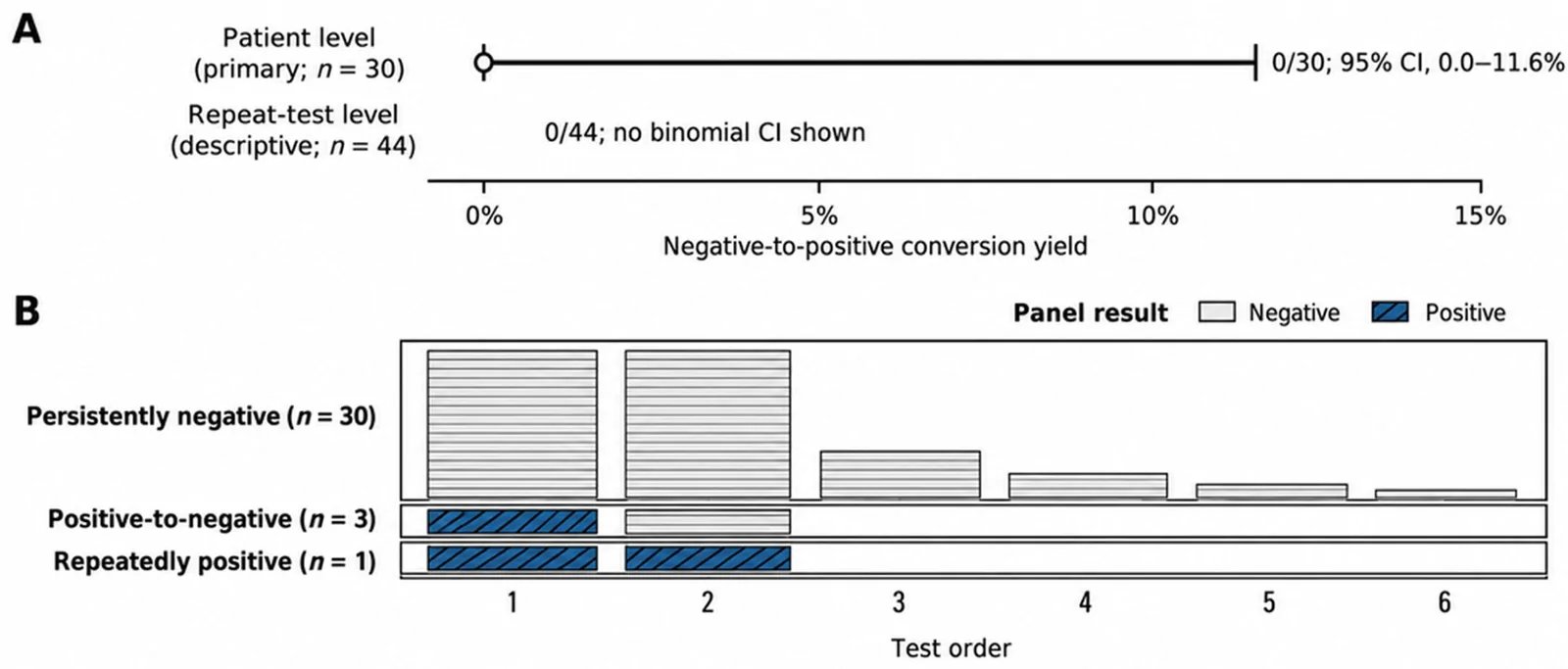
Incremental target-detection yield and patient-level ME-panel result sequences. (**A**) The patient-level point estimate and Clopper–Pearson exact 95% CI for negative-to-positive conversion after a negative first-observed (index) ME-panel result are shown (0/30; 95% CI, 0.0–11.6%). Across the same patients, none of 44 repeat tests detected a new on-panel target; this repeat-test-level count is descriptive and is shown without a binomial CI. (**B**) Chronological ME-panel result sequences among 34 repeat-tested patients; each row is one patient and columns indicate chronological test order. All 30 patients with an index-negative panel remained persistently negative; among four patients with an index-positive panel, three had a subsequent negative result and one remained positive for the same cytomegalovirus target. Gray tiles indicate negative and dark hatched tiles indicate positive results. Abbreviations: CI, confidence interval; ME, meningitis/encephalitis. Alt text: Two-panel figure. Panel (**A**) shows a 0% patient-level negative-to-positive conversion estimate in 30 patients with an exact 95% confidence interval of 0.0% to 11.6%, and zero new detections in 44 repeat tests. Panel (**B**) shows chronological test sequences: 30 patients remained negative, three changed from positive to negative, and one remained positive for the same target.

Among the four patients with an index-positive panel, three converted to negative on subsequent testing and one remained positive for the same cytomegalovirus target on repeat testing. Thus, the only positive repeat-panel episode represented persistent detection of an already identified target rather than a newly detected on-panel pathogen. Across all 34 repeat-tested patients, the observed result sequences were persistently negative in 30 (88.2%), positive-to-negative in three (8.8%), and repeatedly positive in one (2.9%); no negative-to-positive conversion or other mixed sequence was observed (Figure 2).

### 3.4. Exploratory Within-Patient Changes in CSF Cellular and Biochemical Markers

Of the 74 episodes in the persistently negative cohort, 68 (91.9%) were linked to a CSF cellular result within 48 h. The median absolute interval between PCR specimen collection and CSF specimen receipt was 3.88 h (IQR, 1.19–12.64; range, 0.04–40.74 h). Of the 68 linked episodes, 49 (72.1%) were linked within 12 h, 66 (97.1%) within 24 h, and 55 (80.9%) occurred on the same calendar day. CSF receipt preceded PCR collection in 24 episodes and followed PCR collection in 44 episodes. Paired first and last linked measurements were available for 28 patients for CSF WBC and for 27 patients each for protein and glucose. Paired chemistry measurements were unavailable for one of the 28 patients with paired WBC measurements.

CSF WBC decreased from a median of 30.5 cells/µL (IQR, 10.8–277.5) at the first measurement to 3.5 cells/µL (IQR, 1.0–20.5) at the last. The median within-patient change was −29.5 cells/µL (bootstrap 95% CI, −184.0 to −16.5), with a decrease in 24 of 28 patients, no change in one, and an increase in three (Wilcoxon signed-rank *p* < 0.001; FDR q < 0.001). CSF protein decreased from 74.8 mg/dL (0.748 g/L; IQR, 47.1–179.9 mg/dL [0.471–1.799 g/L]) to 53.4 mg/dL (0.534 g/L; IQR, 40.3–102.5 mg/dL [0.403–1.025 g/L]), with a median change of −14.7 mg/dL (−0.147 g/L; bootstrap 95% CI, −37.1 to 0.4 mg/dL [−0.371 to 0.004 g/L]); protein decreased in 18 patients and increased in nine, but the change was not statistically significant either before or after FDR correction (*p* = 0.056; q = 0.084). CSF glucose was essentially unchanged, from 68.0 mg/dL (3.77 mmol/L; IQR, 45.5–82.0 mg/dL [2.53–4.55 mmol/L]) to 66.0 mg/dL (3.66 mmol/L; IQR, 48.0–93.5 mg/dL [2.66–5.19 mmol/L]), with a median change of −4.0 mg/dL (−0.22 mmol/L; bootstrap 95% CI, −10.0 to 4.0 mg/dL [−0.56 to 0.22 mmol/L]); glucose decreased in 16 patients, was unchanged in one, and increased in 10 (*p* = 0.990; q = 0.990) (Table 4; Figure 3).

Restricting linkage to within 24 h excluded two of the 68 linked episodes, both of which had served as the final eligible measurements for two patients in the primary analysis. For each affected patient, the latest remaining eligible measurement became the final value. Because both patients retained at least two eligible measurements, the paired sample sizes remained 28 for WBC and 27 each for protein and glucose. The final paired value changed for all three markers in these patients; nevertheless, the median WBC change and direction-of-change counts remained unchanged, and the WBC decrease remained statistically significant. Under the 24-h linkage definition, WBC decreased from a median of 30.5 to 3.5 cells/µL, with a median paired change of −29.5 cells/µL (bootstrap 95% CI, −184.0 to −16.5; 24 decreased, one unchanged, and three increased; *p* < 0.001; FDR q < 0.001). Protein showed a nominal decrease before multiplicity correction (paired *n* = 27; *p* = 0.036), but did not remain statistically significant after FDR adjustment (q = 0.053); glucose remained nonsignificant (paired *n* = 27; *p* = 0.446; q = 0.446) (Supplementary Table S1, Panel A).

Additional temporal sensitivity analyses yielded consistent WBC findings. When CSF values linked to the index panel and the first repeat panel were compared, paired WBC measurements were available for 26 patients at a median panel interval of 7.85 days (IQR, 5.16–12.70 days). Median WBC decreased from 27.5 to 3.5 cells/µL, with a median paired change of −23.5 cells/µL (bootstrap 95% CI, −128.0 to −3.0); WBC decreased in 21 patients, was unchanged in one, and increased in four (*p* < 0.001; FDR q = 0.002). Protein and glucose were not significant in this analysis. Among 24 patients whose complete observed panel sequence occurred within 30 days, paired WBC measurements were available for 22; WBC decreased in 19 patients, with a median paired change of −23.5 cells/µL (bootstrap 95% CI, −61.0 to −8.5; FDR q < 0.001). Among 21 patients whose complete observed sequence occurred within 14 days, paired WBC measurements were available for 19; WBC decreased in 17 patients, with a median paired change of −26.0 cells/µL (bootstrap 95% CI, −208.0 to −14.0; FDR q = 0.001).

Protein decreased significantly in the 30- and 14-day restricted analyses but not in the primary or index-to-first-repeat analyses. Wilcoxon signed-rank comparisons for glucose were nonsignificant across all sensitivity analyses (Supplementary Table S1, Panels B–D). For glucose in the 14-day complete-sequence analysis, the bootstrap interval for the median paired change excluded zero, whereas the Wilcoxon signed-rank test was nonsignificant; because these procedures address different statistical quantities, the Wilcoxon-based inferential conclusion remained nonsignificant (Supplementary Table S1, Panel D). In the targeted RBC-exclusion sensitivity analysis, one of the 28 paired patients was excluded because an RBC count was missing at either the first or last measurement, and 11 were excluded because the RBC count exceeded 500 cells/µL at either time point, leaving 16 paired patients. Median CSF WBC decreased from 24.0 cells/µL (IQR, 1.8–112.0) at the first measurement to 2.0 cells/µL (IQR, 0.8–6.3) at the last measurement. The median paired change was −22.5 cells/µL (bootstrap 95% CI, −93.0 to −2.0); WBC decreased in 13 patients, was unchanged in one, and increased in two (Wilcoxon signed-rank *p* = 0.008) (Supplementary Table S1, Panel E).

## 4. Discussion

Three laboratory-level observations emerged from this study. First, among the complete cohort of 228 patients with a first-observed index-negative ME panel, patients who subsequently underwent repeat testing had higher index-panel-linked CSF WBC counts and protein concentrations than those with no subsequent repeat test recorded. These differences provide laboratory context for observed repeat-testing patterns but do not establish why clinicians selected individual patients for retesting. Second, among the 30 index-negative patients who were retested, no additional on-panel target was observed across 44 repeat tests. Third, paired CSF WBC values decreased during serial laboratory assessment among persistently negative patients. These data address laboratory-level incremental on-panel detection and serial CSF change and do not establish that CNS infection was absent, that repeat testing was clinically unnecessary, or that the WBC change represented treatment response or recovery.

The overall episode positivity of 12.1% was similar to the 12.2% reported in a tertiary-hospital stewardship cohort of 712 patients [17], although yield varies with patient selection, age distribution, immune status, testing algorithms, and local pathogen epidemiology [5,6,16]. A consecutive series of 4199 CSF samples reported 7.5% pathogen detection [23], and positivity has also varied across Korean cohorts with different populations and testing practices [24,25]. In the present cohort, the only positive repeat-panel episode represented persistent detection of the same cytomegalovirus target already found on the index panel; no repeat test introduced a newly detected on-panel pathogen. This patient-level longitudinal reconstruction distinguishes the study from analyses that treat each order as an independent event.

No negative-to-positive conversion was observed in the primary patient-level analysis (0/30; exact 95% CI, 0.0–11.6%), and the finding was unchanged when repeat testing was restricted to 14 or 30 days. These temporal restrictions reduce, but do not eliminate, concern that widely separated tests represented distinct clinical events. The small primary denominator (*n* = 30) limits precision. Although the observed point estimate was 0%, the exact 95% CI extends to 11.6%, so the data remain compatible with a nonzero conversion rate of potential clinical importance and cannot exclude modest incremental yield in a larger population. For context, with 30 independent patients, a true conversion probability of approximately 9.5% would provide a 95% chance of observing at least one conversion, whereas a true probability of approximately 5.2% would provide an 80% chance. Accordingly, the results do not justify a universal no-repeat rule and do not define a patient-level retesting threshold.

The CSF findings provide complementary, not competing, laboratory information. Median first linked WBC was 30.5 cells/µL and declined to 3.5 cells/µL at the final linked assessment, with decreases in 24 of 28 paired patients. Proposed stewardship algorithms have used pleocytosis to enrich ME-panel positivity [6,17], but safe thresholds may differ by immune status, and clinically significant detections can occur in immunocompromised patients despite minimal pleocytosis [26]. Because immune status and ordering indications were unavailable here, the present WBC findings should not be used to define a retesting threshold. Rather, they show that paired CSF WBC values changed in this observed cohort while panel results remained negative; they do not identify the cause of that change or establish treatment response or clinical recovery.

From a laboratory perspective, three diagnostic actions are analytically distinct: (1) repeating the same syndromic target set, (2) serially reassessing CSF cellular and biochemical findings, and (3) pursuing targeted or off-panel microbiological testing when the clinical differential remains broad. The present data directly describe the first two actions at the laboratory level: no incremental on-panel detection was observed, while paired CSF WBC values decreased. They do not evaluate the third action because culture, targeted PCR, off-panel testing, final diagnoses, and outcomes were not available, nor can they determine whether repeat ordering was clinically appropriate for an individual patient. This distinction is particularly important when clinical suspicion persists despite a negative syndromic panel.

A target-specific exception deserves emphasis. Early CSF sampling can yield a false-negative herpes simplex virus result [10], and current microbiology guidance supports additional targeted testing when herpes simplex encephalitis remains strongly suspected [11]. A large consecutive ME-panel series also reported reduced sensitivity for herpes simplex virus 1 and recommended additional testing in appropriate clinical contexts [23]. These recommendations address a target-specific diagnostic concern and should not be conflated with automatic repetition of the entire syndromic panel. Neither an initial negative panel nor repetition of the same panel substitutes for targeted testing when the clinical hypothesis requires it.

The neurological literature provides a more directly relevant context for repeat CSF evaluation. Guidelines and cohort studies of bacterial meningitis describe repeat lumbar puncture as a selective rather than routine procedure, generally driven by lack of expected clinical responses, diagnostic uncertainty, or specific patient groups [13,15]. In infants with meningitis, repeat CSF sampling has been used to assess bacteriologic clearance and evolving CSF parameters [27,28]. These studies address different populations and endpoints from the present analysis, but they reinforce that repeat CSF assessment is indication-dependent and should not be equated with automatic repetition of the same syndromic molecular panel.

The serial CSF WBC decrease remained statistically significant across the primary comparison and multiple sensitivity analyses. It remained significant with a 24-h PCR–CSF linkage restriction, at the first repeat assessment, in complete sequences restricted to 30 or 14 days, and after excluding paired patients whose first or last CSF specimen had an RBC count >500 cells/µL. The RBC-exclusion sensitivity analysis retained only 16 paired patients and therefore provides limited precision despite the directionally consistent finding. Protein was nonsignificant in the primary analysis and varied across sensitivity analyses, whereas glucose remained nonsignificant throughout; these biochemical findings should be considered exploratory. The WBC decrease should not be interpreted as treatment response or clinical recovery because treatment exposure, symptom trajectory, final diagnosis, and outcomes were unavailable. Prior studies of repeated CSF assessment in bacterial meningitis and infant cohorts nevertheless illustrate that serial CSF findings and repeat pathogen detection can provide different types of information [15,27,28].

This study has several strengths. Testing episodes were reconstructed chronologically at the patient level and quantified against the full tested population, an adjudicated same-specimen duplicate was excluded, and patient-level inference was separated from descriptive repeat-test counts. Uncertainty around the zero-event estimate was explicitly quantified. Molecular outcomes were analyzed separately from serial CSF markers, marker-specific pairing preserved available data, and robustness was assessed across multiple temporal, linkage, and RBC-exclusion definitions.

The study also has important limitations. It was retrospective and single-center, and the primary index-negative repeat cohort included only 30 patients; the zero-event exact 95% CI therefore remains wide enough to include a clinically meaningful nonzero conversion rate. The additional comparison between index-negative patients with and without a subsequent repeat test recorded at this institution during the study period was post hoc and observational. In the multiplicity sensitivity analysis, only the WBC and protein comparisons remained below q < 0.05; the age and sex differences should therefore be regarded as descriptive. Differences in baseline laboratory findings may reflect age, case mix, clinician selection, or other unmeasured clinical factors and should not be interpreted as causal determinants or clinical predictors of repeat ordering. Repeat-testing status was based on ME-panel tests recorded at this institution during the study window. Observation time after the index panel therefore varied among patients, and repeat testing performed at other institutions or after the study period was not captured. Consequently, some patients classified as having no recorded repeat test may have undergone repeat testing outside the observed institutional record. Clinical indications for repeat testing, symptom onset and disease stage at sampling, antimicrobial or antiviral exposure before or between lumbar punctures, immune status, imaging, culture, targeted or off-panel molecular results, final diagnoses, and outcomes were unavailable. We also could not determine whether a repeat lumbar puncture was related to a clinically adjudicated traumatic or iatrogenic circumstance. Consequently, the study cannot determine why panels were reordered, whether clinical suspicion persisted, or whether alternative infectious or noninfectious diagnoses were subsequently established. In particular, the proportions of persistently panel-negative patients ultimately diagnosed with off-panel infections such as tuberculosis or noninfectious disorders such as autoimmune encephalitis could not be estimated. The findings should therefore be interpreted as laboratory-level longitudinal evidence on incremental on-panel detection and serial CSF change, not as a clinical effectiveness study or proof that infection was absent.

Additional limitations relate to measurement and linkage. The 48-h linkage definition could introduce a temporal mismatch, although 97.1% of linked episodes were within 24 h and the WBC result remained significant under the 24-h restriction. Manual Neubauer-chamber counts are less precise at low cell concentrations, and many final values were near the lower reportable range. CSF RBC correction was not applied in the primary analysis; the targeted RBC-exclusion sensitivity analysis retained only 16 patients and should therefore be interpreted cautiously. The RBC threshold was a laboratory sensitivity criterion rather than a clinical adjudication of traumatic lumbar puncture. Intermediate measurements were not modeled, and regression to the mean may have contributed [29]. Larger multicenter studies that combine longitudinal laboratory data with verified ordering indications, targeted/off-panel microbiological results, treatment exposure, immune status, and clinical outcomes are needed to define when repeated syndromic testing adds value.

## 5. Conclusions

In this single-center retrospective laboratory-record cohort, patients with a first-observed index-negative ME panel who subsequently underwent repeat testing had higher index-panel-linked CSF WBC counts and protein concentrations than those with no subsequent repeat test recorded, providing laboratory context for observed repeat-testing patterns. Among the 30 index-negative patients who underwent repeat testing, no negative-to-positive conversion was observed (exact 95% CI, 0.0–11.6%), while paired CSF WBC values decreased during serial laboratory assessment. The small repeat-tested cohort and absence of data on clinical indications for repeat testing, treatment exposure, immune status, and final diagnoses preclude inference that repeat testing is generally unnecessary or that serial CSF changes reflect clinical recovery. Persistently negative ME-panel results do not exclude CNS infection. These laboratory findings cannot determine whether repeat testing is appropriate for an individual patient.

## Figures and Tables

**Table 1:** Construction and characteristics of the repeat-testing cohort.

Characteristic	Value
Overall cohort	
Eligible ME-panel episodes	313
Unique patients	265
Age at first observed panel, median (IQR), years	38.0 (1.0–64.0)
Male sex, *n* (%)	157 (59.2)
Panel-positive episodes, *n* (%)	38 (12.1)
Patients tested once, *n* (%)	231 (87.2)
Patients undergoing repeat testing, *n* (%)	34 (12.8)
Repeat-tested patients	
Episodes among repeat-tested patients	82
Repeat tests among repeat-tested patients	48
Age at index panel, median (IQR), years	55.5 (40.5–58.8)
Male sex, *n* (%)	26 (76.5)
Index panel positive, *n* (%)	4 (11.8)
Sequence length, 2/3/4/5/6 tests, *n*	26/5/1/1/1 patients
Index-to-first-repeat interval, median (IQR; range), days	7.65 (5.52–11.48; 0.36–81.77)
Consecutive-test interval, median (IQR; range), days	7.30 (6.03–11.40; 0.36–81.77)
Index-to-final interval, median (IQR; range), days	9.03 (6.62–14.31; 1.91–93.32)
Repeat tests within 14 days, *n* (%)	31/48 (64.6)
Repeat tests within 30 days, *n* (%)	40/48 (83.3)
Persistently negative cohort	
Patients	30
Episodes	74
Repeat tests after an index-negative result	44
Age at index panel, median (IQR), years	54.5 (40.5–62.0)
Male sex, *n* (%)	23 (76.7)
Sequence length, 2/3/4/5/6 tests, *n*	22/5/1/1/1 patients
Index-to-first-repeat interval, median (IQR), days	7.30 (5.16–12.33)
Consecutive-test interval, median (IQR), days	7.14 (5.87–11.52)
Index-to-final interval, median (IQR), days	8.41 (6.62–14.83)
Observed negative-to-positive conversions	0/30 patients; 0/44 tests

Percentages for patients tested once, repeat-tested patients, panel-positive episodes, and overall sex use the full cohort as the denominator. Characteristics under the repeat-tested and persistently negative sections use the corresponding patient-level cohort as the denominator. All 30 repeat-tested patients with an index-negative panel remained negative during their available within-institution sequences; therefore, the repeat-tested index-negative cohort and the persistently negative longitudinal cohort were numerically identical in this dataset (*n* = 30). The 0/44 repeat-test count is descriptive because tests within patients are not independent. Abbreviations: IQR, interquartile range; ME, meningitis/encephalitis.

**Table 2:** Characteristics of patients with a first-observed index-negative ME-panel result according to subsequent repeat-testing status.

Characteristic	No Repeat Test Recorded	Repeat Testing	*p* Value
Patients, *n*	198	30	—
Age, median (IQR), years	32.0 (0.0–64.0)	54.5 (40.5–62.0)	0.047
Male sex, *n* (%)	110 (55.6)	23 (76.7)	0.030
Index-panel-linked CSF WBC, cells/µL, *n*; median (IQR)	184; 4.0 (1.0–15.0)	27; 28.0 (8.5–262.0)	<0.001
Index-panel-linked CSF protein, mg/dL, *n*; median (IQR)	183; 52.4 (29.5–79.1)	26; 73.4 (46.7–160.0)	0.004
Index-panel-linked CSF glucose, mg/dL, *n*; median (IQR)	183; 60.0 (50.0–73.0)	26; 70.0 (49.5–82.0)	0.271

*p* values were obtained using two-sided Wilcoxon rank-sum tests for continuous variables and Fisher’s exact test for sex. As a multiplicity sensitivity analysis across the five post hoc comparisons, Benjamini–Hochberg FDR adjustment yielded q = 0.059 for age, q = 0.0504 for sex, q < 0.005 for CSF WBC, q = 0.010 for CSF protein, and q = 0.271 for CSF glucose. CSF marker values were restricted to measurements linked to the first-observed index panel; measurements linked only to subsequent repeat panels were not substituted when an index-panel-linked value was unavailable. Denominators vary because index-panel-linked CSF measurements were not available for all patients. These exploratory comparisons describe laboratory context and do not establish clinical indications or causal predictors of repeat ordering.

**Table 3:** Patient-level incremental target-detection yield, temporal sensitivity analyses, and panel-result sequences among repeat-tested patients.

Analysis	No. of Patients or Tests	Observed Outcome	Proportion, % (Exact 95% CI Where Applicable)
Primary incremental target-detection analysis			
Negative-to-positive conversion, patient level (primary)	30 patients	0	0.0 (0.0–11.6)
Negative-to-positive conversion, repeat-test level (descriptive)	44 repeat tests	0	0.0
Temporal sensitivity analyses			
Within 14 days, patient level	25 patients	0	0.0 (0.0–13.7)
Within 14 days, repeat-test level	27 repeat tests	0	0.0
Within 30 days, patient level	28 patients	0	0.0 (0.0–12.3)
Within 30 days, repeat-test level	36 repeat tests	0	0.0
Panel-result sequence among all repeat-tested patients			
Persistently negative	34 patients	30	88.2
Negative-to-positive conversion	34 patients	0	0.0
Positive-to-negative	34 patients	3	8.8
Repeatedly positive	34 patients	1	2.9
Other mixed sequence	34 patients	0	0.0

Exact Clopper–Pearson 95% CIs were calculated only for patient-level estimates. Repeat-test-level counts were descriptive because multiple tests within the same patient were not independent. In temporal sensitivity analyses, denominators included patients or repeat tests occurring within the specified interval from the index panel. Sequence percentages were calculated among all 34 repeat-tested patients. Abbreviations: CI, confidence interval;

**Table 4:** Within-patient changes in CSF cellular and biochemical markers among persistently negative patients.

Marker	Paired *n*	First, Median [IQR]	Last, Median [IQR]	Median Paired Change [95% CI]	Dec/Unch/Inc, *n*	*p* Value	FDR q
CSF WBC, cells/µL (×10^6^/L)	28	30.5 [10.8–277.5]	3.5 [1.0–20.5]	−29.5 [−184.0 to −16.5]	24/1/3	<0.001	<0.001
CSF protein, mg/dL (g/L)	27	74.8 (0.748) [47.1–179.9 (0.471–1.799)]	53.4 (0.534) [40.3–102.5 (0.403–1.025)]	−14.7 (−0.147) [−37.1 to 0.4 (−0.371 to 0.004)]	18/0/9	0.056	0.084
CSF glucose, mg/dL (mmol/L)	27	68.0 (3.77) [45.5–82.0 (2.53–4.55)]	66.0 (3.66) [48.0–93.5 (2.66–5.19)]	−4.0 (−0.22) [−10.0 to 4.0 (−0.56 to 0.22)]	16/1/10	0.990	0.990

Primary table values use the 48-h PCR–CSF linkage definition. Paired change was calculated as the last value minus the first value. *p* values were obtained using two-sided Wilcoxon signed-rank tests; CIs for median paired changes used 20,000 patient-level percentile bootstrap resamples; FDR q values used the Benjamini–Hochberg procedure. For protein and glucose, each table cell reports the conventional-unit value followed in parentheses by the SI-unit value; the bracketed interval is shown in the same unit order. CSF WBC values are numerically identical in cells/µL and ×10^6^/L and are therefore not duplicated. Abbreviations: CI, confidence interval; CSF, cerebrospinal fluid; Dec, decreased; FDR, false discovery rate; Inc, increased; IQR, interquartile range; Unch, unchanged; WBC, white blood cell count.

## Data Availability

The data presented in this study are available on request from the corresponding author. The data are not publicly available due to privacy and institutional restrictions.

## Supplementary Materials

The following supporting information can be downloaded at https://www.mdpi.com/article/10.3390/microorganisms14102310/s1, Table S1: Post hoc sensitivity analyses of within-patient changes in cerebrospinal fluid markers among persistently negative patients; Table S2: Distribution of detected targets among 38 ME-panel-positive episodes.

## Abbreviations

The following abbreviations are used in this manuscript:

CI	confidence interval
CNS	central nervous system
CSF	cerebrospinal fluid
FDR	false discovery rate
IQR	interquartile range
ME	meningitis/encephalitis
PCR	polymerase chain reaction
RBC	red blood cell
WBC	white blood cell
