# Supplementary material for: Repeat Meningitis/Encephalitis Syndromic Panel Testing After an Index-Negative Result: A Retrospective Laboratory-Record Study
Source: Microorganisms. 2026 Oct 10;14(10):2310. doi: 10.3390/microorganisms14102310 (PMC13707547; doi:10.3390/microorganisms14102310)
Supplement: Supplementary file 1 [file microorganisms-14-02310-s001.zip › microorganisms-4510719-supplementary.pdf]

Supplementary Materials

Supplementary Table S1. Post hoc sensitivity analyses of within-patient changes in cerebrospinal fluid markers among persistently negative patients

Marker	Paired n	First, median [IQR]	Comparison, median [IQR]	Median paired change [95% CI]	Dec / Unch / Inc, n	P value	FDR q
**Panel A. PCR–CSF linkage restricted to within 24 h**							
CSF WBC, cells/μL (×10⁶/L)	28	30.5 [10.8–277.5]	3.5 [1.0–16.5]	−29.5 [−184.0 to −16.5]	24 / 1 / 3	<0.001	<0.001
CSF protein, mg/dL (g/L)	27	74.8 (0.748) [47.1–179.9 (0.471–1.799)]	55.3 (0.553) [40.3–95.8 (0.403–0.958)]	−14.7 (−0.147) [−37.1 to 0.4 (−0.371 to 0.004)]	18 / 0 / 9	0.036	0.053
CSF glucose, mg/dL (mmol/L)	27	68.0 (3.77) [45.5–82.0 (2.53–4.55)]	56.0 (3.11) [44.5–84.5 (2.47–4.69)]	−4.0 (−0.22) [−10.0 to 1.0 (−0.56 to 0.06)]	17 / 1 / 9	0.446	0.446
**Panel B. Index panel vs first repeat panel**							
CSF WBC, cells/μL (×10⁶/L)	26	27.5 [6.3–264.0]	3.5 [1.0–33.8]	−23.5 [−128.0 to −3.0]	21 / 1 / 4	<0.001	0.002
CSF protein, mg/dL (g/L)	25	74.8 (0.748) [46.3–180.0 (0.463–1.800)]	55.3 (0.553) [40.2–104.0 (0.402–1.040)]	−7.3 (−0.073) [−18.6 to 1.1 (−0.186 to 0.011)]	15 / 1 / 9	0.236	0.354
CSF glucose, mg/dL (mmol/L)	25	68.0 (3.77) [48.0–82.0 (2.66–4.55)]	71.0 (3.94) [50.0–93.0 (2.77–5.16)]	−4.0 (−0.22) [−11.0 to 4.0 (−0.61 to 0.22)]	16 / 0 / 9	0.647	0.647
**Panel C. Complete observed panel sequence within 30 days (24 eligible patients)**							
CSF WBC, cells/μL (×10⁶/L)	22	24.5 [6.3–189.0]	2.0 [1.0–9.0]	−23.5 [−61.0 to −8.5]	19 / 1 / 2	<0.001	<0.001
CSF protein, mg/dL (g/L)	22	73.4 (0.734) [49.0–99.8 (0.490–0.998)]	48.2 (0.482) [38.3–88.2 (0.383–0.882)]	−15.8 (−0.158) [−34.0 to −3.5 (−0.340 to −0.035)]	16 / 0 / 6	0.010	0.016
CSF glucose, mg/dL (mmol/L)	22	74.0 (4.11) [59.0–90.2 (3.27–5.01)]	69.0 (3.83) [51.0–93.8 (2.83–5.21)]	−7.0 (−0.39) [−14.0 to 1.0 (−0.78 to 0.06)]	16 / 0 / 6	0.537	0.537
**Panel D. Complete observed panel sequence within 14 days (21 eligible patients)**							
CSF WBC, cells/μL (×10⁶/L)	19	27.0 [16.5–232.0]	2.0 [1.0–13.0]	−26.0 [−208.0 to −14.0]	17 / 0 / 2	<0.001	0.001
CSF protein, mg/dL (g/L)	19	85.2 (0.852) [60.2–140.0 (0.602–1.400)]	53.4 (0.534) [39.0–95.8 (0.390–0.958)]	−17.5 (−0.175) [−49.3 to −1.1 (−0.493 to −0.011)]	14 / 0 / 5	0.016	0.024
CSF glucose, mg/dL (mmol/L)	19	68.0 (3.77) [56.5–87.5 (3.14–4.86)]	56.0 (3.11) [50.5–93.5 (2.80–5.19)]	−8.0 (−0.44) [−14.0 to −1.0 (−0.78 to −0.06)]	14 / 0 / 5	0.507	0.507
**Panel E. First-to-last WBC analysis after excluding patients with an RBC count >500 cells/μL in either paired specimen**							
CSF WBC, cells/μL (×10⁶/L)	16	24.0 [1.8–112.0]	2.0 [0.8–6.3]	−22.5 [−93.0 to −2.0]	13 / 1 / 2	0.008	—

Comparison denotes the last linked measurement in Panel A, the first repeat measurement in Panel B, and the last measurement in Panels C–E; paired change = comparison − first. The 24-h restriction replaced the primary-analysis final episode for two patients with the latest remaining eligible measurement; both retained ≥2 eligible measurements, so paired n was unchanged. Panel E excluded one patient with missing RBC data at either paired time point and 11 with RBC >500 cells/μL (>500 ×10⁶/L) in either paired specimen; because it comprised one targeted WBC comparison, FDR adjustment was not applied. P values are two-sided asymptotic Wilcoxon signed-rank tests without continuity correction. CIs are 20,000-resample patient-level percentile bootstrap CIs; Benjamini–Hochberg FDR correction was applied separately within Panels A–D. Bootstrap CIs estimate median paired change, whereas Wilcoxon tests the signed-rank distribution, so their inferential conclusions may differ. Overlapping patient subsets are robustness checks, not independent replications. q

values for Panels A–D were computed from unrounded P values. Protein and glucose cells show conventional units followed by SI units in parentheses, with bracketed intervals in the same order; WBC values are numerically identical in cells/μL and $\times 10^6$/L and are not duplicated. Abbreviations: CI, confidence interval; CSF, cerebrospinal fluid; Dec, decreased; FDR, false discovery rate; Inc, increased; IQR, interquartile range; RBC, red blood cell count; Unch, unchanged; WBC, white blood cell count.

Supplementary Table S2. Distribution of detected targets among 38 ME-panel-positive episodes

Pathogen class	ME-panel target	All positive episodes, n (%)	Index-panel-positive episodes, n	Repeat-panel-positive episodes, n
Viral	Varicella-zoster virus (VZV)	12 (31.6)	12	0
Viral	Enterovirus	8 (21.1)	8	0
Viral	Herpes simplex virus 2 (HSV-2)	7 (18.4)	7	0
Viral	Cytomegalovirus (CMV)	3 (7.9)	2	1
Viral	Human herpesvirus 6 (HHV-6)	2 (5.3)	2	0
Viral	Herpes simplex virus 1 (HSV-1)	0 (0.0)	0	0
Viral	Human parechovirus	0 (0.0)	0	0
Bacterial	*Streptococcus agalactiae*	2 (5.3)	2	0
Bacterial	*Escherichia coli* K1	1 (2.6)	1	0
Bacterial	*Haemophilus influenzae*	1 (2.6)	1	0
Bacterial	*Listeria monocytogenes*	1 (2.6)	1	0
Bacterial	*Streptococcus pneumoniae*	1 (2.6)	1	0
Bacterial	*Neisseria meningitidis*	0 (0.0)	0	0
Fungal	*Cryptococcus neoformans/gattii*	0 (0.0)	0	0
Total	Positive episodes	38 (100.0)	37	1

Percentages were calculated using the 38 ME-panel-positive episodes as the denominator. All positive episodes contained a single detected target; no multiple-target detections were identified. The index-panel-positive category denotes positivity on the first panel recorded during the study period. The single repeat-panel-positive episode represented persistent detection of the same cytomegalovirus target observed on the index panel. Abbreviation: ME, meningitis/encephalitis.
